# Unilateral upper and lower limb ischemia mimics stroke: a case report

**DOI:** 10.1186/s13256-023-04240-1

**Published:** 2024-02-03

**Authors:** Monika Péčová, Jakub Benko, Martin Jozef Péč, Tomáš Bolek, Tatiana Hurtová, Juraj Sokol, Ján Staško, Matej Samoš, Marián Mokáň

**Affiliations:** 1 Oncology Centre, Teaching Hospital Martin, Kollárova 2, 036 01 Martin, Slovakia; 2 Department of Hematology and Transfusion Medicine, National Centre of Hemostasis and Thrombosis, Jessenius Faculty of Medicine in Martin, Comenius University in Bratislava, Kollárova 2, 036 01 Martin, Slovakia; 3 Department of Internal Medicine I., Jessenius Faculty of Medicine in Martin, Comenius University in Bratislava, Kollárova 2, 036 01 Martin, Slovakia; 4 Department of Cardiology, Teaching Hospital Nitra, 950 01 Nitra, Slovakia; 5 Department of Infectology and Travel Medicine and Department of Dermatovenerology, Jessenius Faculty of Medicine in Martin, Comenius University in Bratislava, Kollárova 2, 036 01 Martin, Slovakia; 6 Division of Acute and Interventional Cardiology, Department of Cardiology and Angiology II., Mid-Slovakian Institute of Heart and Vessel Diseases (SÚSCCH, a.s.), 974 01 Banská Bystrica, Slovakia

**Keywords:** Paresthesia, Acute limb ischemia, Stroke, Stroke mimics

## Abstract

**Background:**

Although stroke and acute limb ischemia seem easily distinguishable by anamnesis and physical examination, symptoms may overlap and sometimes mislead the examiner. Such a situation can arise in the occurrence of unilateral neurological symptoms affecting the upper and lower limbs at the same time. As timely diagnosis and a correct therapeutic intervention are crucial to prevent irreversible damage in both diseases, knowledge of the possibility of one disease mimicking the other is essential. We present a unique case of acute unilateral upper and lower limb ischemia mimicking an acute stroke.

**Case presentation:**

A 69-year-old Caucasian patient with known atherosclerotic risk factors was admitted to the emergency department with a suspected stroke with unilateral paresthesia. After a comprehensive examination of the patient with the need for repeated reevaluation and a negative brain computed tomography scan, acute left-sided upper and lower limb ischemia was eventually diagnosed. The patient underwent surgical revascularization of the upper and lower limbs with a satisfactory result and was discharged from the hospital after a few days.

**Conclusion:**

It is of utmost importance to always stay alert for stroke mimics, as overlooking can lead to severe complications and delay adequate therapy. Our case shows that persistent diagnostic effort leads to successful treatment of the patient even on rare occasions, as is the acute unilateral upper and lower limb ischemia.

## Background

Acute limb ischemia (ALI) and stroke are severe conditions that significantly threaten patients’ health. ALI is defined as a sudden decrease in limb perfusion with a potential risk of limb amputation. Limb ischemia is considered acute if the duration of symptoms is less than 2 weeks. Major causes include embolism, thrombosis, aneurysm, dissection, or traumatic vessel injury. The typical presentation of ALI is characterized by the well-known 5 Ps—pain, pulselessness, pallor, paresthesia, and paralysis. All these symptoms are easily detected by physical examination [1]. In the absence of one or more symptoms of ALI, an incorrect evaluation of this condition may occur.

Strokes are among the most common diseases affecting people of all ages. It is the second most common cause of death, accounting for 11% of all deaths worldwide [2] and the world’s leading cause of disability. There are two main etiologies of stroke: reduction or interruption of blood flow to the brain (ischemic stroke) or, in 20–30% of cases, bleeding [3]. A stroke is a deliberate emergency that presents as a focal neurological deficit and requires immediate treatment. A crucial intervention for the prognosis and outcomes of patients is shortening the door-to-needle time—the shorter the time, the more significant the benefit of early revascularization [4]. Noncontrast computed tomography is the first step in evaluating suspicion of stroke in patients due to its ubiquitous availability and relatively quick imaging time [5]. Ischemic stroke mimics can be responsible for up to 20% of clinically diagnosed acute ischemic strokes, and the rate of thrombolysis of mimics can be as high as 17% [6].

This report presents a rare case of a patient admitted to the emergency department because of unilateral paresthesia and clinical suspicion of acute ischemic stroke.

## Case presentation

**Fig. 1:**
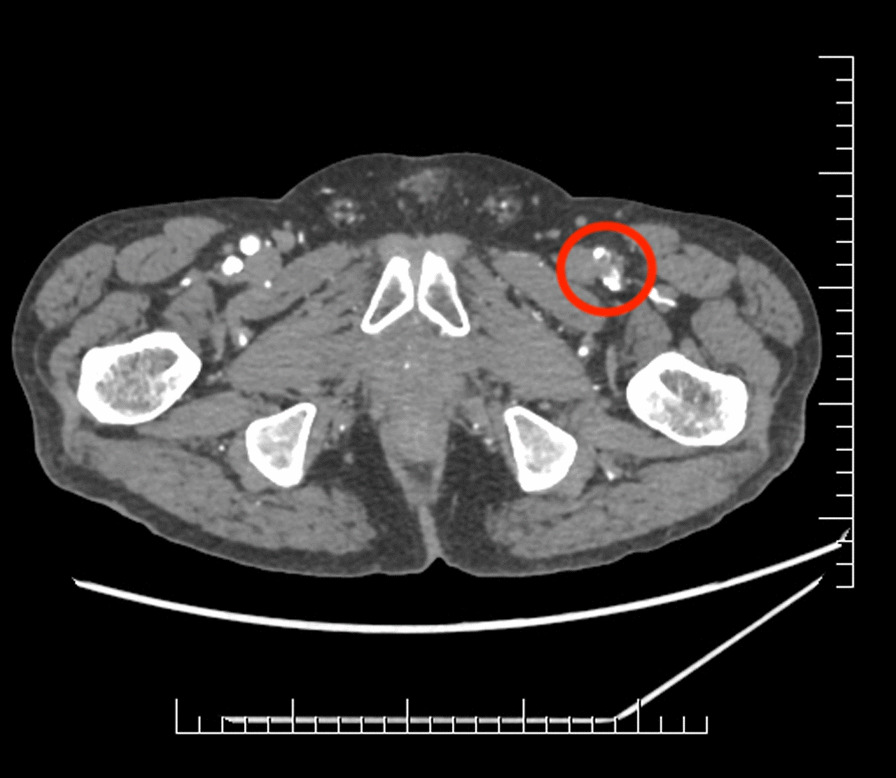
CT angiography showing a saddle-shaped thrombus (in the red circle) in the bifurcation of the left femoral communis artery

**Fig. 2:**
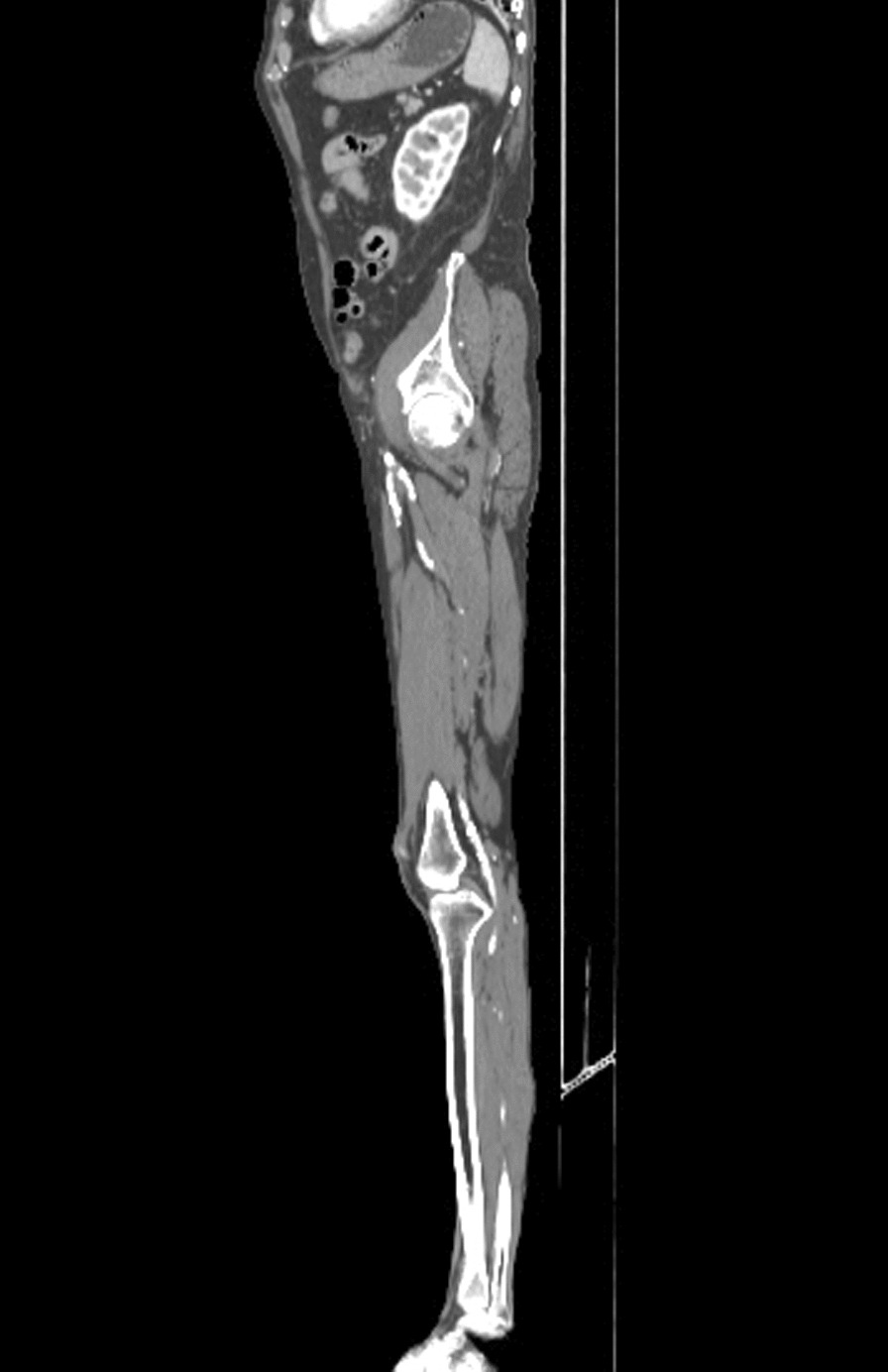
CT angiography showing the thrombus affecting the superficial and deep femoral arteries on the left side

A 69-year-old Caucasian male patient was examined at the emergency department because of numbness in the left upper and lower extremities. The duration of the symptoms was about 2 hours. The past medical history included ischemic heart disease, arterial hypertension, atrial fibrillation, dyslipidemia, and pancreatic lipomatosis. The patient’s medication included a beta blocker, an angiotensin receptor blocker, an alpha sympatholytic, a statin, thyroxine supplementation after total thyroidectomy, and a vitamin K antagonist (VKA) as prevention of systemic embolization. The admission international normalized ratio (INR) value was 1.64. On presentation, the patient had a heart rate of 105 bpm. His blood pressure was 180/100 mmHg, his respiratory rate was 14 beats per minute, and he was fully conscious and oriented and without fever. The neurological examination found paresthesia and mild left upper and lower limb paresis. The left lower limb also showed other signs of ischemia, as pallor and pulselessness were present. We proceeded with a brain computed tomography (CT) scan with angiography, which showed no signs of ischemia or bleeding. The CT angiography showed a 60% stenosis of the C3 part of the right internal carotid artery. These findings did not elucidate the patient’s symptoms, so a CT angiography of the lower left limb was performed. The CT scan showed a saddle-shaped thrombus in the bifurcation of the left femoral communis artery, interfering with the superficial and deep femoral arteries and causing their occlusion (Fig. 1 and 2). Another finding during the CT examination was a 70% stenosis of the left popliteal artery. Afterward, another thorough physical examination of the upper limb was executed, and the finding clinically corresponded to ALI IIb according to the Rutherford classification. The patient underwent an embolectomy of the left brachial, ulnar, radial, femoral, and deep femoral arteries with a complete resolution of symptoms. The hospitalization was uneventful, and the patient was discharged after 6 days. The anticoagulation therapy was changed to dabigatran. The patient was regularly checked up during a 1-year period with no medical problems. His adherence was high, and he was satisfied with oral dosing of anticoagulation drugs.

## Discussion

The efforts to revascularize as soon as possible often lead to a misdiagnosis of stroke. Pohl *et al*. analyzed 61 studies with 62,664 patients whose mimic rate was 24.8%. The most common types of mimics were peripheral vestibular dysfunction, toxic/metabolic etiology, seizure, functional disorder, and migraine. Stroke mimics are more common at a younger age. Affected patients tend to have lower blood pressure values, fewer atherosclerotic risk factors, are predominantly female, and have less severe symptoms than ischemic stroke patients [7].

We present a rare cause of a stroke mimic—unilateral upper and lower acute limb ischemia. The patient presented in this case report developed ALI despite treatment with a VKA. Additional laboratory tests demonstrated ineffective anticoagulation. Verma *et al*. presented a case report of a patient with acute thrombotic occlusion of the subclavian artery presenting as a stroke mimic with early recognition of ALI and successful arterial thrombectomy and vascular repair leading to full recovery [8]. Umoh *et al*. described a patient initially diagnosed with monoparesis of the right leg because of a stroke involving the left anterior cerebral artery. Shortly, the patient was found to have gangrene after developing acute limb ischemia [9]. The case report from authors Lee *et al*. reminds us that ischemic stroke and acute limb ischemia can coincide [10]. Stroke was eventually excluded in our patient by the therapeutic effect of the performed embolectomy. The unique clinical picture of our patient was caused by severe systemic embolization caused by ineffective anticoagulation. Past studies showed that thromboembolism affects the lower limb four times more often than the upper limb. Therefore, the more common stroke mimic should be the lower limb monoparesis [11].

## Conclusion

We are presenting a very rare stroke mimic, which was not described before. To the best of our knowledge, this is the first report about simultaneous unilateral upper and lower limb ischemia that mimics a common subcortical stroke. Time and rapid identification are crucial in prompt and correctly chosen treatment. Physicians should be cautious and keep in mind this rare diagnosis. Overlooked ALI could lead to grave complications such as gangrene, permanent disability, and death. Another issue is the possible mismanagement, resulting in side effects of systemic thrombolysis if the stroke mimic is not recognized.

## Data Availability

The authors confirm that the data supporting the findings of this study are available within the article.
